# Developmental Trajectories of MIDUS: History and Evolving Design Innovations of Team Science

**DOI:** 10.1093/geroni/igaf122.1536

**Published:** 2025-12-31

**Authors:** David Almeida

**Affiliations:** The Pennsylvania State University, University Park, Pennsylvania, United States

## Abstract

The Midlife in the United States (MIDUS) study has continuously evolved since its launch in 1995 with initial funding from The MacArthur Foundation. Originally designed to examine how psychosocial factors shape health and aging, MIDUS was groundbreaking in its inclusion of population-based daily diary studies, which provided novel insights into how everyday stressors accumulate to influence long-term well-being. With the first P01-funded expansion from the National Institute on Aging (NIA), MIDUS shifted beyond surveys to integrate biomarkers, cognitive testing, and affective neuroscience measures, marking a pivotal transition toward biopsychosocial integration. The addition of a diverse Milwaukee sample strengthened its generalizability, while new methodological innovations linked socioeconomic conditions to neurobiological health outcomes. In 2011, the MIDUS Refresher was introduced to address emerging societal challenges, such as the Great Recession, and to enhance statistical power for studying intersectional factors like race, gender, and socioeconomic status. More recently, the study has focused on maintaining sample representativeness by re-engaging past participants, a strategy that allows researchers to assess the long-term effects of early vulnerability markers on aging trajectories. As MIDUS moves into its next phase- the second and fourth waves of collection for the refresher and core samples, it continues to exemplify team science, fostering interdisciplinary collaboration to explore the complex interplay of daily experiences, social inequalities, and biological aging. By continuously adapting to new scientific priorities, MIDUS remains at the forefront of lifespan health research, providing a powerful model for studying adult development in a changing world.

